# Rationale and Design of the Türkiye Heart Failure (TURK‐HF) Registry

**DOI:** 10.1002/clc.70353

**Published:** 2026-05-21

**Authors:** Umut Kocabas, Selda Murat, Tarık Kıvrak, Bektaş Murat, Cihan Altın, Cennet Yıldız, Şeyda Günay Polatkan, Selma Akdeniz Oskay, Zeynep Ulutaş, Duygu İnan, Nuran Günay, Dilay Karabulut, Fahri Çakan, Veysel Yavuz, Isil Ergin, Uğur Önsel Türk, İstemihan Tengiz, The TURK‐HF Registry Investigators, Veysel Ozan Tanık, Türkan Seda Tan Kürklü, Ali Nail Kaya, Mevlüt Demir, Taner Şen, Çağatay Tunca, Gülsüm Meral Yılmaz Öztekin, Meltem Altınsoy, Ümit Yaşar Sinan, Cengiz Şabanoğlu, Mehmet Kaplan, Emre Özçalık, Işık Tekin, Oğuzhan Birdal, Mustafa Doğduş, Anıl Şahin, Örsan Deniz Urgun, Emin Erdem Kaya, Fulya Avcı Demir, Mustafa Yenerçağ, Abdullah Tunçez, Umut Karabulut, Halil İbrahim Tanrıseven, İbrahim Halil Özdemir, Melih Öz, Cemre Turgul, Atakan Şengöz, Nurullah Çetin, Fahri Er

**Affiliations:** ^1^ Department of Cardiology Başkent University Izmir Hospital Izmir Türkiye; ^2^ Department of Cardiology Eskisehir Osmangazi University Eskisehir Türkiye; ^3^ Department of Cardiology Firat University Medical School Elazig Türkiye; ^4^ Department of Cardiology Eskisehir City Hospital Eskisehir Türkiye; ^5^ Department of Cardiology, Faculty of Medicine, Medical Point Hospital Izmir University of Economics Izmir Türkiye; ^6^ Department of Cardiology Bakirkoy Dr Sadi Konuk Training and Research Hospital Istanbul Türkiye; ^7^ Department of Cardiology, Faculty of Medicine Bursa Uludag University Bursa Türkiye; ^8^ Department of Cardiology Medicana International Izmir Hospital Izmir Türkiye; ^9^ Department of Cardiology İnönü University Malatya Türkiye; ^10^ Department of Cardiology Başakşehir Çam and Sakura City Hospital İstanbul Türkiye; ^11^ Department of Cardiology Ümraniye Training and Research Hospital İstanbul Türkiye; ^12^ Department of Cardiology Çorlu State Hospital Tekirdag Türkiye; ^13^ Department of Cardiology Akhisar Mustafa Kirazoglu State Hospital Manisa Türkiye; ^14^ Department of Public Health, Faculty of Medicine Ege University Izmir Türkiye

**Keywords:** heart failure, outcome, real‐world data, registry, treatment, Türkiye

## Abstract

**Aims:**

The Türkiye Heart Failure (TURK‐HF) registry aims to identify the sociodemographic and clinical characteristics, management strategies, and outcomes of patients with heart failure (HF) and to assess the implementation of evidence‐based HF therapies in Türkiye.

**Methods:**

The TURK‐HF registry is a national, multicenter, prospective, observational study of unselected patients with HF regardless of ejection fraction. A total of 46 investigators from 38 centers in 22 cities across Türkiye participated in the registry. The study investigators will gain access to the electronic case report form via www.turkhf.com website using their usernames and passwords. The baseline assessment of the patients will include sociodemographic data, primary care access information, frailty assessment, health‐related quality of life questionnaire, HF‐related information, medical history and comorbidities, physical examination findings, electrocardiographic and echocardiographic data, H_2_FPEF score calculation, laboratory results, and medical and device‐based HF therapies. The management strategies and potential complications of patients hospitalized with acute HF will be systematically documented. Follow‐up data will be collected at regular outpatient visits every 6 months with a margin of error of 1 month. The clinical endpoints of the TURK‐HF registry were cardiovascular or all‐cause mortality, HF‐related hospitalizations, clinician‐interpreted outcomes, patient‐reported outcomes, and surrogate endpoints, either alone or in combination. The TURK‐HF registry was registered at ClinicalTrials.gov (ID: NCT06707220).

**Conclusion:**

The TURK‐HF registry offers comprehensive and distinctive insights into contemporary HF clinical characteristics, diagnostic methods, treatments, and outcomes. This registry has the potential to influence implementation strategies, clinical research, and public policies across Türkiye.

## 1 Introduction

Heart failure (HF) is a public health concern associated with higher rates of hospitalization, mortality, and healthcare resource utilization [1]. The incidence of HF in Europe is approximately five per 1000 person‐years in adults, with a prevalence of HF estimated to be between 1% and 2% of the population [2]. However, the prevalence of HF is predicted to rise due to the aging of the population, improved survival rates after myocardial infarction and cancer, advances in pharmacological and device‐based HF therapies, and the greater utilization of heart transplantation over the last decades [1]. Despite these advances, the prognosis of HF remains worse than that of many cancer types, with a 5‐year mortality rate varying from 45% to 60% in adult patients [3].

The proposed terminology based on left ventricular ejection fraction (LVEF) measurement defines “heart failure with reduced ejection fraction” (HFrEF) as LVEF of ≤ 40%, “heart failure with mildly‐reduced ejection fraction” (HFmrEF) as LVEF of 41%–49%, and “heart failure with preserved ejection fraction” (HFpEF) as LVEF ≥ 50% [2]. The main rationale for this terminology is based on clinical trials that have demonstrated beneficial outcomes in patients with HFrEF. Currently, guideline‐directed medical therapies (GDMT), comprising renin‐angiotensin system (RAS) inhibitors, β‐blockers, mineralocorticoid receptor antagonists (MRAs), and sodium−glucose cotransporter‐2 (SGLT‐2) inhibitors, are recommended for patients with HFrEF to reduce the risk of HF hospitalization and death [1, 2]. Notwithstanding compelling evidence from randomized controlled trials and recommendations in current guidelines, the utilization of GDMT among patients with HFrEF remains suboptimal in routine clinical practice [4]. While registry‐based cohort studies have identified numerous patient‐, physician‐, and healthcare system‐related factors associated with the non‐use of GDMT, the discrepancy between current guidelines and clinical practice has not yet been fully elucidated [5, 6].

In contrast, HFpEF is distinct from HFrEF and represents a heterogeneous clinical syndrome. There is a lack of knowledge regarding the pathophysiological mechanisms, comorbidity burden, and phenotypes of HFpEF [7]. Consequently, our understanding of this complex disease remains limited, and the diagnosis and/or treatment of patients with HFpEF remains a significant challenge. In contrast to patients with HFrEF, treatment options for patients with HFpEF are limited. Historically, numerous randomized clinical trials encompassing RAS inhibitors, β‐blockers, and MRAs have failed to show efficacy in patients with HFpEF [1, 2]. In recent years, several promising studies have been conducted. The results of phase 3 clinical trials with SGLT‐2 inhibitors and finerenone have demonstrated favorable outcomes in patients with HFmrEF and/or HFpEF [8, 9, 10]. However, there are still limited real‐world data on the clinical phenotypes and comorbidity burden in patients with HFpEF and the prognostic impact of these novel treatments.

Acute HF is characterized by the sudden onset of symptoms, either de novo HF or due to the worsening of previously diagnosed HF (WHF) [1, 2]. The term WHF is used to describe the progression of disease in patients with chronic HF that requires hospitalization or unplanned emergency department visits. WHF is recognized as an acute deterioration phase in the natural course of the disease, indicating poor prognosis [11]. Despite its clinical importance, WHF remains inadequately defined, and its management remains challenging. Current WHF data are obtained from randomized controlled trials with a highly selected HF population. Although the prognostic significance of WHF events during the course of the disease is known, there is a paucity of data on the definition, management, and outcomes of WHF in real‐world patients.

A comprehensive understanding of the sociodemographic, clinical, and biological characteristics of patients with HF in real‐world settings is essential for developing novel strategies to improve patient outcomes. Identifying the barriers to GDMT implementation in a real‐world setting is critical for decreasing the risk of HF hospitalization and mortality [1, 2]. A comprehensive and well‐designed HF registry can determine the clinical characteristics of patients with HF, identify patient‐, physician‐, and healthcare system‐related factors for the non‐use of evidence‐based therapies, and improve the implementation of GDMT [12]. It should be noted that the number of HF registries that enroll patients with de novo HF, chronic HF (outpatients), and acute HF (hospitalized or treated in the emergency department) in all three relevant EF categories (HFrEF, HFmrEF, and HFpEF) is limited. Furthermore, only a few registries have assessed the detailed aspects of guideline adherence, treatment decisions, and both in‐hospital and long‐term outcomes of these patients. Therefore, the Türkiye Heart Failure (TURK‐HF) registry was designed to address this knowledge gap regarding the identification, management, and long‐term prognosis of patients with HF in a real‐world setting. The registry also aimed to document barriers to externalizing current guidelines for routine patient care in the country.

## 2 Methods

### 2.1 Design

The TURK‐HF registry is a national, multicenter, prospective, observational study of unselected patients with HF, regardless of LVEF (HFrEF, HFmrEF, or HFpEF), who presented with de novo, chronic, or acute HF requiring hospitalization and/or emergency department admission. This study was approved by the ethics committee (project no. KA23/429–E‐91694447‐604.01‐336905) and will be conducted according to the principles of the Declaration of Helsinki. All patients will provide written informed consent for participation. The TURK‐HF registry was registered at ClinicalTrials.gov (ID: NCT06707220).

### 2.2 Study Sites and Participant Physicians

**Figure 1:**
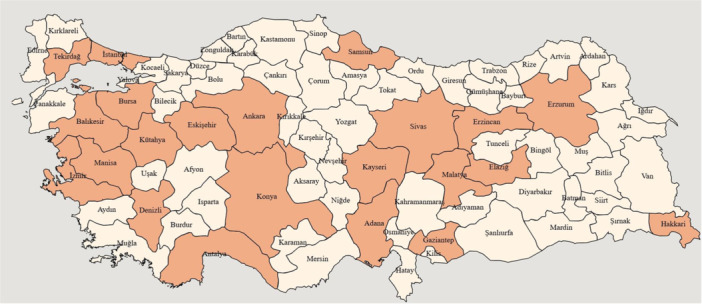
Geographical distribution of 22 cities in Türkiye that participated in the TURK‐HF registry.

The TURK‐HF registry will be conducted in seven distinct geographical regions in Türkiye. The study sites were identified according to the Nomenclature of Territorial Units for Statistics (NUTS) Level 1 classification system to reflect the population distribution of geographical regions [13]. Detailed data on the cities, NUTS regions, and study sites are presented in Table 1 and Figure 1. To reflect the characteristics of health providers, the study sites were selected based on the projected distribution of health provider subgroups, as presented in the Health Statistics Yearbook [14]. The participant physicians were offered participation by the project coordinator. It was made clear that participation was voluntary and that no financial compensation would be provided for participation. A total of 46 investigators from 38 centers (16 university hospitals, 13 education and research hospitals or city hospitals, 4 state hospitals, and 5 private hospitals) in 22 cities across Türkiye participated in the study (Table 1 and Figure 1). The diagnosis of HF, initiation, up‐titration, and maintenance of HF treatments, disease management, and treatment‐related adverse events and complications are beyond the scope of this registry and are the responsibility of the participating physicians.

**Table 1:** Nomenclature of Territorial Units for Statistics (NUTS) Level 1 regions, cities, and study sites of the TURK‐HF registry.

NUTS Level 1 region	City	Study site
Aegean (TR3)	Izmir	Baskent University, Faculty of Medicine, Department of Cardiology
	Izmir	Medicana International Izmir Hospital, Department of Cardiology
	Izmir	Izmir University of Economics, Faculty of Medicine, Department of Cardiology
	Izmir	Ege University, Faculty of Medicine, Department of Public Health
	Kütahya	Kutahya Health Sciences University, Faculty of Medicine, Department of Cardiology
	Manisa	Manisa Celal Bayar University, Department of Cardiology, Manisa
	Manisa	Manisa City Hospital, Department of Cardiology, Manisa
	Manisa	Akhisar Mustafa Kirazoglu State Hospital, Department of Cardiology, Manisa
	Denizli	Pamukkale University, Department of Cardiology, Denizli
Istanbul (TR1)	Istanbul	Istanbul University‐Cerrahpasa Institute of Cardiology, Department of Cardiology
	Istanbul	Bakirkoy Dr. Sadi Konuk Training and Research Hospital, Department of Cardiology
	Istanbul	Dr. Siyami Ersek Training and Research Hospital, Department of Cardiology
	Istanbul	Umraniye Training and Research Hospital, Department of Cardiology
	Istanbul	Basaksehir Cam and Sakura City Hospital, Department of Cardiology
	Istanbul	Acibadem International Hospital, Department of Cardiology
West Marmara (TR2)	Tekirdağ	Çorlu State Hospital, Department of Cardiology
	Tekirdağ	Tekirdağ City Hospital, Department of Cardiology
	Balıkesir	Bandırma Onyedi Eylül University, Department of Cardiology
East Marmara (TR4)	Bursa	Bursa Uludag University, Faculty of Medicine, Department of Cardiology
	Eskişehir	Eskisehir Osmangazi University, Faculty of Medicine, Department of Cardiology
	Eskişehir	Eskisehir City Hospital, Department of Cardiology
West Anatolia (TR5)	Ankara	Ankara University, Faculty of Medicine, Department of Cardiology
	Ankara	Ankara Etlik City Hospital, Department of Cardiology
	Ankara	LÖSANTE Hospital, Department of Cardiology
	Konya	Selcuk University, Faculty of Medicine, Department of Cardiology
Mediterranean (TR6)	Antalya	Antalya Training and Research Hospital, Department of Cardiology
	Antalya	Antalya Medical Park Hospital, Department of Cardiology
	Adana	Cukurova State Hospital, Department of Cardiology
Central Anatolia (TR7)	Sivas	Sivas Cumhuriyet University, Faculty of Medicine, Department of Cardiology
	Kayseri	Kayseri Training and Research Hospital, Department of Cardiology
West Black Sea (TR8)	Samsun	Samsun University, Faculty of Medicine, Department of Cardiology
Northeast Anatolia (TRA)	Erzincan	Mengucek Gazi Education and Research Hospital, Department of Cardiology
	Erzurum	Atatürk University, Faculty of Medicine, Department of Cardiology
Central East Anatolia (TRB)	Elazığ	Firat University, Faculty of Medicine, Department of Cardiology
	Malatya	Inonu University, Faculty of Medicine, Department of Cardiology
Southeast Anatolia (TRC)	Gaziantep	Gaziantep Medical Park Hospital, Department of Cardiology
	Gaziantep	Gaziantep City Hospital, Department of Cardiology
	Hakkari	Hakkari State Hospital, Department of Cardiology

### 2.3 Study Population

The only inclusion criterion for the TURK‐HF registry was the presence of HF as determined by the participating physician. The exclusion criteria were age < 18 years and refusal to provide informed consent for registry participation. All patients will be eligible for enrollment, regardless of LVEF (HFrEF, HFmrEF, or HFpEF) or whether they have de novo or chronic HF. All patients will be enrolled in the study, regardless of their clinical status. This encompasses both those presenting for a non‐urgent outpatient visit with stable signs and symptoms and those requiring urgent care for WHF. The objective of this approach was to ensure the representativeness of the study population to the source population to generalize the aim of generalizability of data and externally validate the registry data. WHF is defined as worsening symptoms and signs of HF in patients with chronic HF, requiring hospitalization and/or intravenous diuretic therapy [11]. All study participants provided written informed consent.

### 2.4 Data Capture, Storage, Security, and Quality Management

The TURK‐HF registry data will be collected using an electronic case report form (e‐CRF) integrated into an electronic data capture system. The e‐CRF application was developed using the PHP application development language and ran on the Oracle MySQL database. The application is hosted on the service provider's cloud servers in a data center provided by a third party. Daily backups are created in accordance with the backup, restore, and disaster recovery procedures. The application is web‐based and accessible to assigned users with different authorization levels. The study investigators will gain access to the e‐CRF via the www.turkhf.com website using their username and password. Participant physicians who enter data belonging to the centers can only access the data they have entered. User management, access to reports, and authority to view all patient data belong to the user with the administrator role. To guarantee the protection of personal data in Türkiye, the Personal Data Protection Regulation (Law No. 6698) was established [15]. For this purpose, the data collected from patients will be pseudonymized, which means that the personal data cannot be attributed to any specific person without additional information. The data were stored in a centralized registry database that was accessible only to authorized personnel and protected by individual passwords.

### 2.5 Baseline Clinical Assessment of Patients

**Figure 2:**
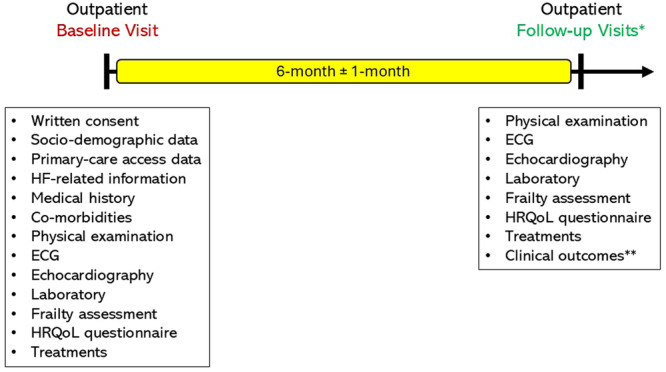
Baseline and follow‐up data elements and time points for patients with chronic heart failure (outpatients) and acute heart failure (hospitalized patients). *Follow‐up data will be collected at regular outpatient visits every 6 months, with a margin of error of 1 month, or via telephone interviews if the patient is not eligible for outpatient visits. **The clinical endpoints of the TURK‐HF registry were cardiovascular or all‐cause mortality, heart failure‐related hospitalizations, clinician‐interpreted outcomes (e.g., New York Heart Association functional class), patient‐reported outcomes (e.g., health‐related quality of life), and surrogate endpoints (e.g., N‐terminal pro‐brain natriuretic peptide levels), either alone or in combination.

The baseline assessment of patients will include sociodemographic data, primary care access information, frailty assessment, health‐related quality of life (HRQoL) questionnaire, HF‐related information, medical history and comorbidities, physical examination findings, electrocardiographic and echocardiographic data, H_2_FPEF score calculation for patients with HFpEF, laboratory results, and medical and device‐based HF therapies (Figure 2).

#### 2.5.1 Sociodemographic Data

To determine the socio‐economic status (SES) and health inequalities, detailed data will be obtained on age, sex, marital status, urban or rural living, housing conditions and household income, education level, and employment status [16]. A detailed approach was adopted to ascertain the SES of individuals in the TURK‐HF Registry. For this purpose, we will question the age, sex, marital status (married or living together, divorced or separated, partner died, or never married), living area (urban or rural), and living conditions (living alone, living only with a partner, living with children and/or grandchildren, or living in a nursing home) of the participants. We classified the education level as no formal education, incomplete primary education, completed primary or secondary education, completed high school, and university graduate. The employment status of the participants was derived from their responses to two questions regarding employment status and profession. We used the classification system of Dr. Korkut Boratav, a Turkish economist, for employment status as follows: an employer with ≥ 10 employees, an employer with 3–9 employees, an employer with ≤ 2 employees, a professional, a white‐collared or office worker, a blue‐collared or skilled employee, a retired individual, an unskilled worker, a farm worker, a marginal worker, an unemployed individual, and a housewife [16]. A series of additional inquiries were posed to retired participants to ascertain their employment status and profession before retirement, determine whether they were still employed after retirement, and, if so, what position they held. Unemployment status does not accurately reflect the real socioeconomic situation of housewives; thus, we asked additional questions about the employment status and profession of the person who contributed the most to the household income of housewives. The participants were asked to choose from extremely poor, poor, fair, high, and extremely high household incomes. Participants were invited to provide information regarding the employment status and profession of their parents and their household income during their period of residence with them. Furthermore, patients will be surveyed using the World Values Survey questionnaire to evaluate their self‐reported health and happiness [17].

#### 2.5.2 Primary Care Access Information

All patients will be asked to provide information regarding the role of their primary care physician in the management of HF. The survey questions regarding primary care access were as follows: (i) Do you know the name of your primary care physician? (ii) Has your primary care physician made any changes to your HF medications? (iii) Did your primary care physician refer you to any specialist because of complaints associated with HF? These survey questions will enable us to obtain real‐world information about the individual and structural determinants of access to primary healthcare and the availability and accessibility of primary healthcare professionals and facilities in Türkiye for patients with HF.

#### 2.5.3 Frailty Assessment

The term “frailty” describes a multidimensional syndrome that leads to a loss of reserves, including physical ability, energy, cognition, and health status. Frailty is a poor prognostic factor that affects more than half of patients with HF [18]. Therefore, we integrated the Canadian Study of Health and Aging Clinical Frailty Scale into the TURK‐HF registry to measure the clinical frailty of patients with HF based on clinical judgment [19, 20, 21]. The Clinical Frailty Scale proposed by the Canadian Study of Health and Aging is a seven‐item scale ranging from 1 (indicating robust health) to 7 (indicating complete functional dependence on others) [19]. Frailty was evaluated at the time of study enrollment by a participant physician using the Canadian Study of Health and Aging Clinical Frailty Scale. This approach is closest to the true meaning of frailty, which is defined as a loss of physiological reserve independent of comorbid conditions or reasons for physical limitation, and it considers cognitive deficits [18, 19].

#### 2.5.4 HRQoL

**Figure 3:**
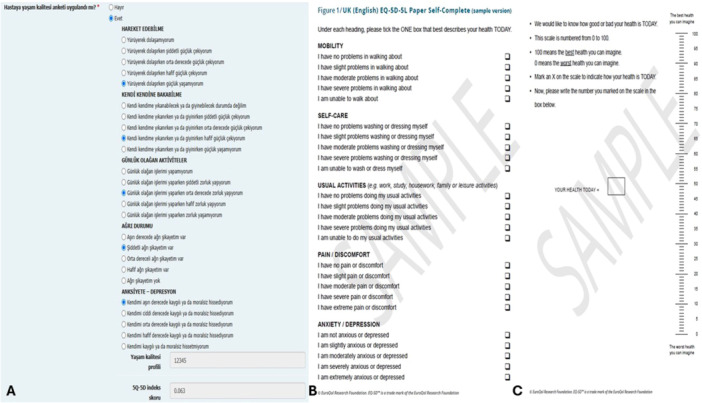
An example of the Turkish version of the EQ‐5D‐5L descriptive system integrated into the electronic case report form in the TURK‐HF registry (A), an example of the English version of the EQ‐5D‐5L descriptive system (B), and an example of THE EQ visual analog scale (C).

HRQoL is defined as an individual's perceived physical and mental health over time, and HF is associated with poor HRQoL [22]. The EQ‐5D‐5L is a widely used generic measure of health status that comprises two components. The first part (the descriptive system) assesses HRQoL in five dimensions, each representing a specific aspect of the patient's HRQoL. The five dimensions are mobility, self‐care, usual activities, pain/discomfort, and anxiety/depression [23]. Each dimension has five response levels (no problems, slight problems, moderate problems, severe problems, and extreme problems/unable). This part of the EQ‐5D‐5L questionnaire provides a descriptive profile that can be used to generate a health state profile. For example, a patient in health state 12345 would have no problems with mobility, slight problems with self‐care (washing or dressing), moderate problems with doing usual activities, severe pain or discomfort, and extreme anxiety or depression (Figure 3A,B). The second part of the questionnaire comprises a visual analog scale, on which the patient rates their perceived health from 0 (representing the worst imaginable health) to 100 (representing the best imaginable health) (Figure 3C). The EQ‐5D‐5L is a valid and reliable instrument for measuring HRQoL and estimating utility values in patients with HFrEF and HFpEF [23, 24, 25, 26, 27]. The TURK‐HF registry was registered with the EuroQol Customer Portal registration number 68900. Accordingly, the EQ‐5D‐5L questionnaire will be administered to patients enrolled in the TURK‐HF registry to determine their HRQoL.

#### 2.5.5 HF‐Related Information, Medical History, and Comorbidities

HF‐related information, comprising the New York Heart Association (NYHA) functional classification, type of HF (HFrEF, HFmrEF, HFpEF, or HF with improved ejection fraction), duration of disease (de novo, < 6 months, or ≥ 6 months), previous history of HF‐related hospitalization or unplanned emergency department visits requiring intravenous diuretic treatment, and etiology of HF, will be obtained during the baseline visit. Participants will be questioned about their medical history, including a history of myocardial infarction, revascularization procedures, heart valve surgery, and left ventricular assist device implantation. Comorbidities include atrial fibrillation, peripheral artery disease, cerebrovascular disease, hypertension, dyslipidemia, diabetes, chronic kidney, lung, and liver disease, obstructive sleep apnea syndrome, anemia, thyroid disease, inflammatory connective tissue diseases, previous history of malignancy, and malignancy treatments (surgery and/or chemotherapy and/or radiation therapy), depression, and dementia will also be recorded.

#### 2.5.6 Physical Examination, Electrocardiography, Echocardiography, and Laboratory Data

Physical examination findings (systolic and diastolic blood pressure, heart rate, height, and weight) of the registry participants will be assessed during the baseline visit. The patients' body mass index will be calculated automatically using an e‐CRF system. Electrocardiographic data, including heart rhythm (normal sinus rhythm, atrial fibrillation or flutter, pacemaker rhythm, or others), presence and type of bundle branch block, and QRS duration; echocardiographic data, including LVEF, left atrium diameter, mitral E/e' ratio, tricuspid regurgitation velocity, estimated systolic pulmonary artery pressure, tricuspid annular plane systolic excursion, and the presence of valvular abnormalities; and laboratory results, including fasting blood glucose, HbA1c, hemoglobin, serum creatinine, sodium, and potassium, alanine aminotransferase, brain natriuretic peptide or N‐terminal pro‐b‐type natriuretic peptide, thyroid stimulant hormone, uric acid, ferritin, transferrin saturation, cancer antigen 125, and urine albumin‐creatinine ratio, will be registered during the first patient visit. The patient's creatinine clearance will be calculated automatically by the e‐CRF system using the Cockcroft−Gault formula [28].

#### 2.5.7 H_2_FPEF Score Calculation

**Figure 4:**
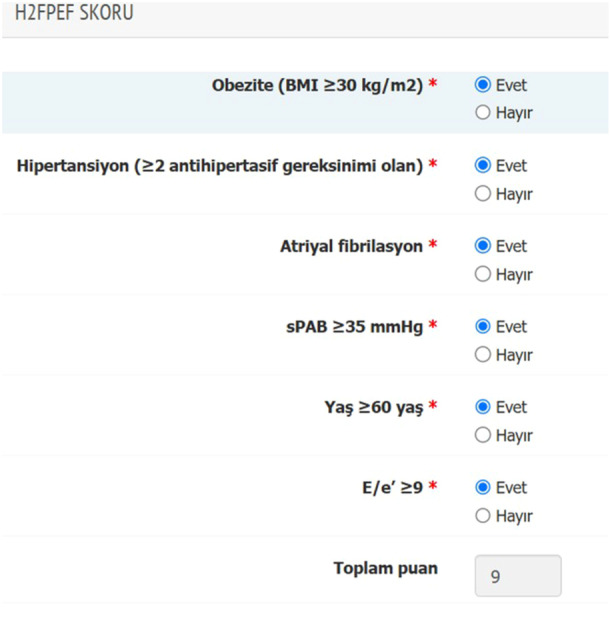
An example of the automatic calculation system integrated into the electronic case report form as a “decision support tool” to calculate the H_2_FPEF score in patients with heart failure with preserved ejection fraction.

It is critical that scoring systems accurately determine the probability of HFpEF to enable clinicians to decide whether HFpEF is present. The validated H_2_FPEF score utilizes six clinical and echocardiographic parameters to accurately distinguish HFpEF from the non‐cardiac causes of dyspnea [29]. A meta‐analysis reported that a high H_2_FPEF score is associated with an increased risk of HF‐related hospitalization and all‐cause mortality in patients with dyspnea and normal LVEF [30]. However, there are conflicting data regarding the use of scoring systems and their diagnostic utility in the Turkish population [31]. Therefore, we integrated an automatic calculation system into the e‐CRF as a “decision support tool” to calculate the H_2_FPEF score in patients with HFpEF (Figure 4).

#### 2.5.8 Medical and Device‐Based HF Therapies

**Figure 5:**
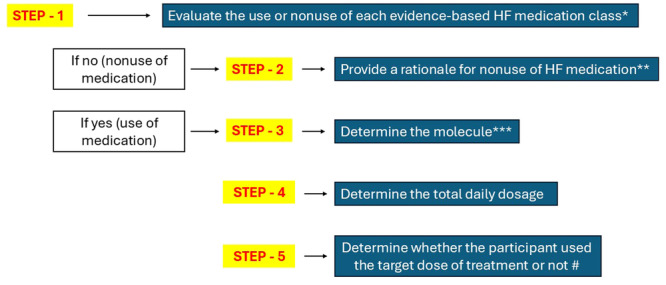
“Treatment assessment algorithm” integrated into the electronic case report form to determine the use or non‐use of guideline‐directed medical therapies by registry participants. Step‐1: Determine the evidence‐based medication classes* (RAS inhibitors, β‐blockers, MRAs, and SGLT‐2 inhibitors) used by the registry participants. Step‐2: In instances where patients were not receiving treatment for HF, the patient and participant physician were asked to provide a rationale** (e.g., symptomatic hypotension for RAS inhibitors, bradycardia for β‐blockers, hyperkalemia for MRAs, or urinary tract infections for SGLT‐2 inhibitors). Step‐3: In instances where patients were receiving treatment for HF, determine the molecule that the participant used*** (e.g., ramipril for RAS inhibitors, bisoprolol for β‐blockers, eplerenone for MRAs, and dapagliflozin for SGTL‐2 inhibitors). Step‐4: Determine the daily dosage of medication that the participant used (e.g., ramipril 2.5 mg once daily, bisoprolol 10 mg once daily, eplerenone 25 mg once daily, and dapagliflozin 10 mg once daily). Step‐5: Determine whether the participant used the target dose of treatment or not# (< 50% of target dose, ≥ 50% to < 100% of target dose, or ≥ 100% of target dose).

We integrated an assessment algorithm into the e‐CRF to assess the use or non‐use of GDMT by registry participants. Within the scope of this algorithm, the following objectives were identified: first, to determine the treatment classes comprising RAS inhibitors, β‐blockers, MRAs, and SGLT‐2 inhibitors that the participant used; second, in instances where patients were not receiving treatment for HF, the patient and participant physician were asked to provide a rationale for this; third, to ascertain which molecule the participant used (e.g., ramipril for RAS inhibitors, bisoprolol for β‐blockers, eplerenone for MRAs, and dapagliflozin for SGTL‐2 inhibitors); fourth, to identify the daily dosage of each molecule that the participant used; and finally, to determine whether the participant used the target dose of treatment or not (< 50% of target dose, ≥ 50% to < 100% of target dose, or ≥ 100% of target dose) (Figure 5). Other medical treatments, including diuretics (loop, acetazolamide, and thiazide diuretics), ivabradine, glucagon‐like peptide‐1 agonists, digoxin, nitrates, statins, antiplatelet and anticoagulants, ferric carboxymaltose, and a previous history of vaccination, and device‐based HF therapies, including cardiac pacemaker, implantable cardioverter‐defibrillator, or cardiac resynchronization therapy, were also assessed.

#### 2.5.9 Additional Clinical Data Collection for Hospitalized Patients With Acute HF

**Figure 6:**
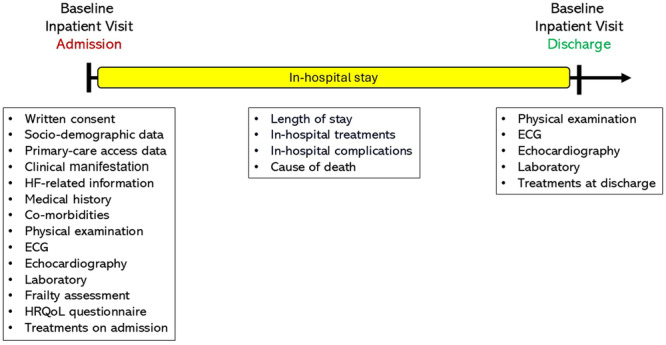
Data elements and time points for hospitalized patients with acute heart failure.

The study timeline for clinical data collection from hospitalized patients with acute HF is presented in the figure. Initially, the participating physicians ascertained the clinical manifestations of acute HF, including acute decompensated HF, acute pulmonary edema, cardiogenic shock, and isolated right ventricular failure [2]. In addition to the sociodemographic and clinical data previously referenced, management strategies will be documented for hospitalized patients diagnosed with acute HF. In‐hospital management strategies encompass oxygen therapy, ventilatory support (noninvasive and/or invasive mechanical ventilation), intravenous treatments (diuretics, inotropes, and/or vasopressors), device therapies (pacemaker, implantable cardioverter‐defibrillator, and cardiac resynchronization therapy), renal replacement therapy, invasive procedures (percutaneous coronary intervention, transcatheter aortic valve replacement, mitral or tricuspid valve transcatheter edge‐to‐edge repair), and mechanical circulatory support devices (intra‐aortic balloon pump, extracorporeal membrane oxygenation, or ventricular assist device therapies) will be registered (Figure 6).

In‐hospital complications, including acute myocardial infarction, cardiogenic shock, ischemic stroke, transient ischemic attack, systemic embolism, deep venous thrombosis, acute pulmonary embolism, atrial and ventricular arrhythmias, second‐ or third‐degree atrioventricular block, acute renal failure, major bleeding, and sepsis, will be documented. During the hospitalization period, if it occurs, the cause of cardiovascular death (WHF, acute myocardial infarction, sudden cardiac death, arrhythmia, stroke, or others) or non‐cardiovascular death (malignancy, systemic infection and sepsis, respiratory failure, renal failure, and others) will also be documented (Figure 6).

A comprehensive evaluation of the patient will be conducted at the time of hospital (or emergency department) admission and again before discharge, encompassing a physical examination, electrocardiographic and echocardiographic data, and laboratory results. We aimed to assess the effects of in‐hospital management strategies on clinical status, physical examination, electrocardiographic, echocardiographic, and laboratory variables. Similarly, we will assess the use of evidence‐based oral HF therapies, including RAS inhibitors, β‐blockers, MRAs, and SGLT‐2 inhibitors, before, during, and after hospitalization. This assessment aimed to determine the in‐hospital optimization of GDMT and its prognostic effectiveness in registry participants (Figure 6).

### 2.6 Follow‐Up Visits

Longitudinal data will be collected at regular outpatient visits every 6 months, with a margin of error of 1 month, or via telephone interviews if the patient is not eligible for outpatient visits (Figure 2). During follow‐up visits, physical examination findings, electrocardiographic and echocardiographic data, laboratory results, and a comprehensive assessment of HF therapies will be obtained. Follow‐up information will be obtained for clinical outcomes (hospitalization for HF and survival) and changes in HF medications. Participants enrolled in the TURK‐HF registry will undergo frailty assessment and, if available, complete the EQ‐5D‐5L questionnaire to determine longitudinal changes in frailty status and HRQoL during follow‐up visits.

### 2.7 Clinical Endpoints

The clinical endpoints of the TURK‐HF registry will reflect clinical “hard” endpoints, including cardiovascular mortality or all‐cause mortality, morbidity outcomes (HF‐related hospitalizations or unplanned emergency department visits requiring intravenous diuretic therapy), clinician‐interpreted outcomes (e.g., NYHA functional class), patient‐reported outcomes (e.g., HRQoL), and surrogate endpoints (e.g., N‐terminal pro‐brain natriuretic peptide levels), either alone or in combination.

The utilization of composite clinical endpoints is a prevalent practice in clinical trials. The time‐to‐first‐event method is the most common approach for analyzing composite endpoints. However, this method is subject to an inherent limitation in that it treats all contributory endpoints equally in terms of their severity. There is growing recognition of the importance of hierarchical composite clinical outcomes and “the win ratio” in cardiology trials [32]. Win ratio analysis prioritizes the most clinically significant component of the composite endpoints by assigning a greater ranking weight to the constituent component [33]. Thus, we will use the win ratio method to analyze the composite outcomes of the TURK‐HF registry.

We will also obtain follow‐up data on classical 3‐point major adverse cardiovascular events, including nonfatal stroke, nonfatal myocardial infarction, and cardiovascular death. Clinical data on hospitalization due to atrial or ventricular arrhythmias, hospitalization due to non‐cardiovascular causes, and left ventricular assist device implantation will be recorded.

### 2.8 Current Situation

The first participant was enrolled in the TURK‐HF registry on January 8, 2025. To date, we have registered 1240 patients with HF within the first 6 months of the study. The initial baseline results of the TURK‐HF registry will be announced after a 1‐year enrollment period. Subsequently, we intend to publish several scientific papers addressing the gaps and potential questions within the HF population.

## 3 Discussion

The TURK‐HF registry will be the first national, large‐scale, multicenter, prospective, and observational study comprising outpatients and hospitalized patients with HF, irrespective of their LVEF, conducted in Türkiye. The primary goal of the present registry is to determine the epidemiology, sociodemographic and clinical characteristics, diagnostic approaches, management strategies, and long‐term prognosis of patients with HF in a real‐world setting. The six fundamental research questions that the TURK‐HF registry aimed to answer are as follows: (i) the impact of SES on the utilization of GDMT and prognosis in patients with HF; (ii) the temporal changes in frailty and HRQoL and its association with subsequent prognosis; (iii) diagnostic approaches, algorithms, and scoring systems for patients with HFpEF; (iv) the comorbidity burden in patients with HFpEF and the phenotypes of HFpEF; (v) the patient, physician, and healthcare system‐related barriers for the suboptimal use of evidence‐based therapies, its prognostic impact, and the strategies to improve the implementation of GDMT; and (vi) in‐hospital management strategies, complications, and prognosis in patients with acute HF.

Previous studies conducted in Western countries have demonstrated that patients with low SES have a higher incidence and prevalence of HF [34, 35]. A published post hoc analysis of the study reported worse clinical outcomes in patients with lower SES [36]. However, the socioeconomic structure of Türkiye differs from that of European, American, and Far Eastern countries, and national data from Türkiye on the impact of SES on the use of GDMT and prognosis in patients with HF are lacking. Therefore, the collection of current data from the real‐life registry is crucial to understand the actual relationship between SES and access to healthcare, receiving GDMT, and prognosis in patients with HF in Türkiye. For the first time, the TURK‐HF registry used the analytic approach described by Boratav to determine the SES of the study participants [16]. As Boratav posits, social classes and individuals' SES are delineated by their relationships within the production process, which are predicated on the control over the means of production. Thus, the TURK‐HF registry design will allow us to obtain detailed data on the participants' control over the means of production, in addition to classical SES variables, including marital status, living area, and education level.

The diagnosis of HFpEF presents challenges due to its multifactorial etiologies and the lack of a definitive diagnostic test. The H_2_FPEF score was created to aid in diagnosing HFpEF. It originated from a study involving 414 patients in the United States with an LVEF of 50% or higher, where the HFpEF diagnosis was confirmed through invasive hemodynamic stress testing [29]. Nonetheless, there is conflicting data concerning the use of scoring systems and their diagnostic utility within the Turkish population. To address this, for the first time, we incorporated an automatic calculation system into the TURK‐HF registry as a “decision support tool” to compute the H_2_FPEF score for patients with HFpEF. This unique approach will provide comprehensive data on the use and diagnostic power of the present scoring system in real‐world patients with HF.

It is widely recognized that patients with HFpEF constitute a heterogeneous cohort, and a “one size fits all” management strategy for HFpEF is inappropriate for this patient population. The clustering strategy to identify distinct phenogroups within populations of patients with HFpEF provides data for patient‐level treatment according to comorbidities and risk factors [37, 38]. A prospective study conducted in the United States identified distinct characteristics among three phenogroups. Phenogroup 1 consisted of younger individuals with lower levels of BNP. Phenogroup 2 demonstrated the highest prevalence of obesity, diabetes mellitus, and obstructive sleep apnea. Conversely, phenogroup 3 comprised the oldest individuals, who were most likely to have chronic kidney disease and exhibited the highest BNP levels [37]. Similarly, a study of the TOPCAT trial participants revealed that phenogroup 1 patients were younger, had a higher prevalence of smoking, and had preserved functional class; phenogroup 2 patients were older with left atrial enlargement and a higher prevalence of atrial fibrillation; and phenogroup 3 patients demonstrated more functional impairment, obesity, diabetes, chronic kidney disease, and concentric LV hypertrophy [38]. To date, clustering strategies and phenogroups within the Turkish population affected by HFpEF have not been studied. The TURK‐HF registry will provide the first national real‐world data on the phenotypes of patients with HFpEF in Türkiye. This initiative will enable personalized treatment strategies for these heterogeneous groups in the era of precision medicine.

Suboptimal implementation of evidence‐based HF treatment, particularly in HFrEF, has been documented in various registries [4, 5, 6, 39, 40]. While most reports have been predominantly descriptive, detailing the percentage usage of fundamental HF medications, they often lack comprehensive information on diagnostic methodologies and additional pertinent medications, devices, and interventions. Well‐structured registries have the potential to collect extensive descriptive data and analyze and quantify the underlying causes of inadequate implementation. This can facilitate the identification of specific patient groups with the most significant unmet needs or areas requiring improvement, thereby enabling targeted initiatives to enhance the implementation. The TURK‐HF registry will provide detailed real‐world data on the management strategies used for patients with HF. For this purpose, we created a unique algorithm and integrated it into the e‐CRF as a “work‐up tool” for the first time in Türkiye. This approach included five consecutive questions about the use or non‐use of GDMT in HF. As outlined in the methods section, we questioned the use of treatment classes and scrutinized the specific molecule prescribed and its daily dosage for each category of evidence‐based HF medication. In this way, we will assess the use or nonuse of the target dose or the maximally tolerated dose of HF medications in patients with HF. Furthermore, we examined the justification for the absence of GDMT utilization, considering factors such as contraindications, intolerability, and clinical inertia. Analyzing the real‐world factors and obstacles hindering the effective implementation of GDMT through the TURK‐HF registry offers a unique opportunity to address these challenges in Türkiye.

## 4 Conclusion

The TURK‐HF registry is the inaugural national, multicenter, prospective HF registry in Türkiye. It is poised to offer comprehensive and distinctive insights into contemporary HF clinical characteristics, diagnostic methods, treatment implementation, and outcomes. This registry has the potential to influence implementation strategies, clinical research, and public policies across Türkiye.

## Conflicts of Interest

The authors declare no conflicts of interest.

## Data Availability

Data sharing is not applicable to this article as no data sets were generated or analyzed during the current study.
